# Overexpression of the Starch Phosphorylase-Like Gene (*PHO3*) in *Lotus japonicus* has a Profound Effect on the Growth of Plants and Reduction of Transitory Starch Accumulation

**DOI:** 10.3389/fpls.2016.01315

**Published:** 2016-08-31

**Authors:** Shanshan Qin, Yuehui Tang, Yaping Chen, Pingzhi Wu, Meiru Li, Guojiang Wu, Huawu Jiang

**Affiliations:** ^1^Key Laboratory of Plant Resources Conservation and Sustainable Utilization, South China Botanical Garden, Chinese Academy of SciencesGuangzhou, China; ^2^University of Chinese Academy of SciencesBeijing, China

**Keywords:** starch phosphorylase, gene expression, starch metabolism, pollen fertility, *Lotus japonicus* L.

## Abstract

Two isoforms of starch phosphorylase (PHO; EC 2.4.1.1), plastidic PHO1 and cytosolic PHO2, have been found in all plants studied to date. Another starch phosphorylase-like gene, *PHO3*, which is an ortholog of *Chlamydomonas PHOB*, has been detected in some plant lineages. In this study, we identified three PHO isoform (*LjPHO*) genes in the *Lotus japonicus* genome. Expression of the *LjPHO3* gene was observed in all tissues tested in *L. japonicus*, and the LjPHO3 protein was located in the chloroplast. Overexpression of *LjPHO3* in *L. japonicus* resulted in a drastic decline in starch granule sizes and starch content in leaves. The *LjPHO3* overexpression transgenic seedlings were smaller, and showed decreased pollen fertility and seed set rate. Our results suggest that *LjPHO3* may participate in transitory starch metabolism in *L. japonicus* leaves, but its catalytic properties remain to be studied.

## Introduction

Starch phosphorylase (α-glucan phosphorylase, PHO; EC 2.4.1.1) catalyzes the reversible transfer of glucosyl units from glucose-1-phosphate to the non-reducing ends of α-1,4-D-glucan chains with the release of phosphate. Two major forms of PHO, the plastidic PHO1 or PHOL (which has a low affinity for glycogen) and the cytosolic PHO2 or PHOH (high glycogen affinity), have been observed across all the higher plants (Wirtz et al., 1980; Kruger and ap Rees, 1983). PHO1 has an additional 78–80 amino acid region (L78 domain) near the middle of the GT1_Glycogen_Phosphorylase domain. The L78 domain in PHO1 has a PEST region which serves as a signal for degradation in sweet potato (Chen et al., 2002; Lin et al., 2012). Mori et al. (1993) suggested that L78 domain in potato PHO1 lowered the affinity of the enzyme for large, branched substrates. Removal of the L70/L80 domain in rice PHO1 did not significantly alter the catalytic and regulatory properties of PHO1 but did affect heat stability (Hwang et al., 2016).

In *Arabidopsis*, mutants lacking PHS1 (plastidic PHO1) have normal patterns of diurnal starch metabolism and no significant changes in starch structure, indicating that this enzyme is not essential for starch synthesis (SS) or degradation. However, the plants display increased sensitivity to drought stress and there is local accumulation of starch around stress-induced lesions (Zeeman et al., 2004). Genetic studies support a role for the PHS1 protein in transitory starch degradation (Malinova et al., 2014). In rice, the loss of PHO1 leads to a reduction in endosperm starch content and shrunken seeds when plants are grown at 20°C, though not when they are grown at 30°C (Satoh et al., 2008). The cytosolic starch phosphorylase PHO2 may be involved, together with the cytosolic transglucosidase DPE2, in the metabolism of cytosolic maltose and heteroglycans formed by starch degradation in leaves (Fettke et al., 2006; Lu et al., 2006). In potato, altered levels of PHO2 mainly affect the molecular properties of the heteroglycans (Fettke et al., 2005). In *Chlamydomonas reinhardtii*, mutation in *STA4*, which encodes the PHOB protein, results in a significant reduction in starch content, the formation of abnormally shaped starch granules containing chain-length modified amylopectin and increased relative amounts of amylase under N-deficiency conditions (Dauvillée et al., 2006). These changes in starch content and structure indicate that PHOB plays a significant role during storage SS.

The ever-growing databases of genomic DNA sequences from “model” and “non-model” plants offer greatly enhanced opportunities to detect previously unknown gene families in higher plants. After comparing gene families involved in starch biosynthetic pathways in *Jatropha curcas* L. with those in *Arabidopsis* and other plants, we found that *J. curcas* L. and some dicots have an ACT domain-containing starch phosphorylase isoform (PHOA or PHO3) which had not previously been reported in higher plants. Phylogenetic analysis suggested that the putative PHO3 proteins form a new subclade with PHOB proteins from the green algae *Ostreococcus lucimarinus* and *C. reinhardtii* (Wu et al., 2015). In the study presented here we explored the function of the *PHO3* gene in *Lotus japonicus*. Our results revealed that the PHO3 protein was located in the chloroplast, and overexpression of the *PHO3* gene decreased starch accumulation in leaves and influenced plant growth and fertility in *L. japonicus*. The results presented here represent the first data on the function of the PHO3 protein subfamily in higher plants.

## Materials and Methods

### Plant Growth and Bacterial Strains

*Lotus japonicus* genotype ‘MG-20’ was used as the wild-type control for phenotypic and genotypic analysis. Seeds were scarified for 10 min in sulfuric acid and planted in vermiculite irrigated with Broughton and Dilworth (B&D) nutrient solution without nitrogen. After planting, seedlings were inoculated with *Mesorhizobium loti* MAFF303099. For testing, all plants were grown in a growth chamber (day/night cycles of 18 h/6 h; temperature 22°C/20°C). For harvesting seeds, seedlings were planted in peat–vermiculite inoculated with *M. loti* MAFF303099 and irrigated with B&D nutrient solution without nitrogen.

### Sequence Retrieval and Analysis

Sequences of PHO proteins were retrieved from GenBank^1^. *Arabidopsis* PHO proteins were used as queries in BLAST searches against the *L. japonicus* genome database^2^. Conserved motifs in PHO proteins were analyzed using the NCBI’s CDD database^3^ (Marchler-Bauer et al., 2015). The plastid transit peptide cleavage site of the PHO proteins was predicted using the program TargetP Server v1.01^4^ (Emanuelsson et al., 1999). For phylogenetic analysis, multiple sequence alignments of PHO amino acid sequences were performed using ClustalW. The tree was constructed using the neighbor-joining (NJ) method and 100 bootstraps in order to group putative full-length PHO amino acid sequences, and the results were displayed with Mega software version 4 (Tamura et al., 2007).

### Plasmid Constructs and Plant Transformation

For construction of the overexpression vector, full length *LjPHOA* cDNA was amplified by RT-PCR using the primers given in Supplementary Table S1. After digestion by restriction enzymes, the cDNA fragments were cloned into the *Kpn* I/*Xba* I sites of pCAMBIA 1302 behind the 35S promoter. The resulting construct was introduced into *Agrobacterium tumefaciens* strain AGL1 by the freeze–thaw procedure. Transformation of *L. japonicus* was carried out according to the method described by Chen et al. (2014).

For subcellular location analysis, the complete coding sequences with the exception of the stop codon were amplified by PCR with the primers given in Supplementary Table S1. After digested by restriction enzymes, the cDNA fragments were cloned into the *Bam* HI*/Xho* I sites of pSAT-EYFP-N1 upstream of the *EYFP* gene. Protoplasts of *Arabidopsis* were isolated and transformed according to the method described by Yoo et al. (2007).

For protein expression in *Escherichia coli*, three fragments, P1 (^Δ41^PHO), P2 (^Δ183^PHO), and P3 (complete CDS) were amplified by PCR with the primers given in Supplementary Table S1. The PCR products were digested and introduced into the pGEX-KG vector at the sites of *Xba* I/*Xho* I (P1and P3) or *Nco* I/*Xho* I (P2) on the 3’-terminus of the GST gene (without stop codon).

### Expression of LjPHO in *E. coli*

Cultures of *E. coli* strain Rosetta containing pGEX-KG (native plasmid), P1, P2, and P3 were grown in Luria–Bertani medium. Overnight cultures were inoculated into fresh medium at a 1:100 dilution and grown at 37°C until the A600 was 0.6. IPTG was added to 0.5 mM and the cultures were grown for 8 h at 22°C. Cells were collected from 400 mL cultures by centrifugation, suspended in one-twentieth culture volume of sonication buffer (50 mM Tris–acetate, pH 7.5, 10 mM EDTA, and 5 mM DTT), and broken by sonication. Lysates were cleared by centrifugation at 10,000 × *g* for 10 min, and the supernatants (crude enzyme extracts) were used for subsequent analyses. The proteins were separated by 7.5% SDS-PAGE. Gels were stained with Coomassie Brilliant Blue R-250 (Jiang et al., 2003).

The recombinant proteins were purified using the GST⋅BIND^TM^ Resin (Novagen^®^, cat. No. 70541)^5^ following the manufacturer’s instructions. GST-LjPHO3 recombinant protein fractions were selected after SDS-PAGE analysis and pooled together. The proteins were precipitated by slowly added two volumes of saturated ammonium sulfate solution pre-chilled at 4°C. After precipitation by centrifugation, the pellet was resuspended with 1 ml of 25 mM HEPES-NaOH buffer (pH 7.0; containing 10% glycerol). The protein solution dialyzed against 3 × 400 mL of the 25 mM HEPES-NaOH buffer at 4°C for 24 h. After clarification by centrifugation the pure enzyme preparation was stored at -80°C until used for analysis. For enzyme assay, both crude enzyme extracts and purified recombinant proteins were tested, respectively (Zeeman et al., 2004). The phosphorylase b from rabbit muscle (P6635, sigma) was used as a positive control to ensure the reaction system for the activity determination was adopted.

### RNA Isolation and qRT-PCR

Samples used for expression analysis were: leaves, roots, stems, and nodules from 3-week-old seedlings; whole unexpanded flowers; young siliques 1–1.5 cm in length; and developing seeds 15–20 days after flowering. Total RNA was extracted from tissues of *L. japonicus* using an RNeasy Plant Mini Kit (QIAGEN)^6^ following the manufacturer’s instructions, and the isolated RNA was treated with RNase-free DNase I (Roche)^7^. First-strand cDNA was synthesized from 2 μg RNA using M-MLV reverse transcriptase (Promega^8^) according to the manufacturer’s instructions. Primer pairs for the PHO genes were designed by the Primer3 software^9^. Pairs of primers are selected which can give specific DNA amplification by PCR. *Ubiquitin* (GenBank accession No. AFK37806) was used as a reference gene. The primers are given in Supplementary Table S1. QRT-PCR was performed on a Mini Option real-time PCR system (LightCycler 480). Cycling conditions were as follows: 95°C for 30 s, 95°C for 5 s, 60°C for 20 s, and 72°C for 20 s. The reaction was performed for 40 cycles. The experiment was performed with three biological replicates and average values are presented (Chen et al., 2014).

### Laser Scanning Confocal Microscopy and Electron Microscopy

Fluorescence images were recorded with a laser scanning confocal microscope (LSM510 META, Zeiss^10^). eYFP fluorescence was imaged at an excitation wavelength of 514 nm (30% power), and the emission wavelength of 527 nm. Chlorophyll fluorescence was imaged at an excitation wavelength of 543 nm, and the emission wavelength of 562 nm.

To obtain ultra-thin sections, leaf four or five from the top of the main branch in 10-week seedlings was fixed in 2.5% glutaraldehyde and 2% paraformaldehyde, then dehydrated in an ethanol series and embedded in Spurr resin. Ultra-thin sections (0.1 μm) were stained with 2% uranyl acetate for 1 h and 6% lead citrate for 20 min and observed with an electron microscope (JEM-1010, Jeol^11^; Jiang et al., 2007).

### Analysis of Starch and Soluble Sugars

Leaves (100 mg fresh weight, hand-homogenized using liquid nitrogen) were extracted three times with 1 ml of ethanol (80% v/v) for 10 min at 80°C. The supernatant after each extraction was recovered by centrifugation. After the last extraction, the pellet was washed with 0.5 ml of 80% ethanol. All supernatants were transferred to a test tube and the combined volume was adjusted to 5 ml with 80% ethanol. Soluble sugar content was determined by a colorimetric method with a sulfuric acid-phenol reagent using a sucrose standard curve (DuBois et al., 1951). The remaining ethanol-insoluble residue was extracted twice by suspension in 1 ml purified water and incubation at 100°C for 30 min; the supernatants were recovered by centrifugation. The supernatants were transferred to a test tube and the combined volume was adjusted to 5 ml with purified water. The starch content was measured by a colorimetric method using an iodine solution and calculated according to a potato starch standard curve.

### Preparation of Enzymes from Leaves and Enzyme Measurements

All procedures were performed at 0-4°C. Fresh leaves (100 mg) were hand-homogenized with a glass homogenizer in 500 μL of solution containing 100 mM HEPES-NaOH (pH 7.5), 1 mM EDTA, 5 mM DTT, 10 mM MgCl_2_, 20 mM KCl, and 10% (v/v) glycerol. The homogenate was centrifuged at 12, 000 × *g* for 10 min, and the pellet was washed (250 μL × 2) with the same buffer. The resulting supernatants were used for the preparation of enzymes.

The ADP-glucose pyrophosphorylase (AGPase) assay was carried out according to the method described by Nishi et al. (2001). The SS and branching enzyme (BE) assay was according to the method described by Jiang et al. (2003). The starch phosphorolysis activity of starch phosphorylase was assayed using a continuous assay in the direction of Glc-1-P formation, coupled to the production of NADH (Zeeman et al., 2004). The SS activity of starch phosphorylase was detected on native glycogen-containing zymograms (Dauvillée et al., 2006).

### Observation of Pollen Morphology and Germination

For the observation of pollen morphology and fertility, pollen grains were harvested from fully expanded flowers. After staining with iodine–potassium iodide solution, pollen was viewed with a bright-field microscope. To test the pollen germination rate, pollen grains were dispersed in a solution of 10% sucrose, 0.01% H_3_BO_3_, 0.05% Ca(NO_3_)_2_⋅4H_2_O. After incubating at 37°C for 30 min, germinated pollen grains were viewed with a bright-field microscope.

## Results

### Identification and Characterization of the Putative Starch Phosphorylase Genes in the *L. japonicus* Genome

**FIGURE 1:**
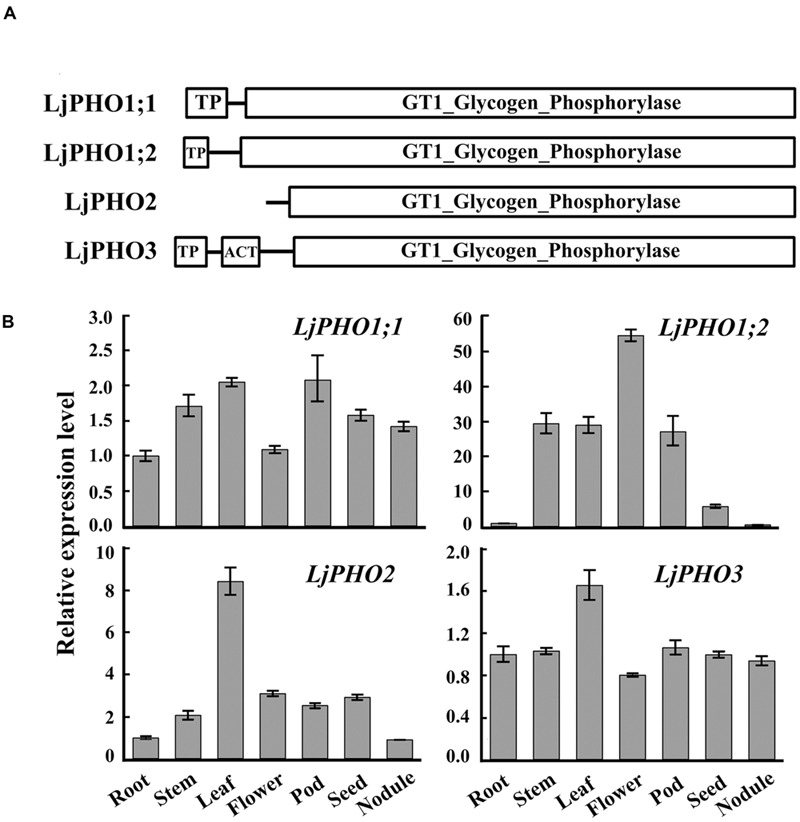
**PHO genes detected in the *Lotus japonicus* genome. (A)** Comparison of the domains found within the predicted amino acid sequences of PHO proteins; **(B)** Expression analysis of the putative *PHO* genes. QRT-PCR of total RNA samples was used to measure putative *PHO* gene expression. Relative expression was normalized to the reference gene *ubiquitin* (internal control). Bars show means ± SD of three biological replicates.

TBLASTN searches of the *L. japonicus* genome^12^ using the amino acid sequences of *Arabidopsis* PHO proteins revealed four putative *PHO* genes. The *L. japonicus PHO* genes were designated *LjPHO1;1* (Lj2g3v1079510), *LjPHO1;2* (Lj0g3v0360239), *LjPHO2* (LjB08M07.90.r, database build 2.5), and *LjPHO3* (Lj6g3v2006830), respectively, on the basis of the unrooted NJ-tree (**Supplementary Figure S1**) constructed from PHO proteins of selected plants. Alignment of the genomic DNA sequences with the cDNA sequences reveals that the *LjPHO* genes contain 15–20 exons separated by 14–19 introns within their coding domain sequences (Supplementary Table S2). *LjPHO2* contains two additional introns in its 5’ untranslated region. All LjPHO proteins contain the GT1_Glycogen_Phosphorylase domain (cd04300). LjPHO1 and LjPHO3, but not LjPHO2, contain a putative plastid-targeting peptide region (TP) as predicted by ChloroP 1.1^13^ (**Figure 1A**). LjPHO3 contains an ACT domain (cl09141) in the N-terminal region. The two LjPHO1 proteins have the L78 region near the middle of the GT1 domain; this region does not exist in the LjPHO2 and LjPHO3 proteins (**Supplementary Figure S2**).

The expression levels of transcripts encoded by the *LjPHO* genes were measured by qRT-PCR in only one developmental stage, including leaf, root, stem, and nodule of 3-week-old plants, and flower, pod and seed of 10-week-old plants of *L. japonicus* MG-20. The results showed that the *LjPHO2* gene was expressed at the highest level in leaves, while *LiPHO1;2* was expressed weakly in roots, seeds, and nodules. The expression levels of *LjPHO1;1* and *LjPHO3* differed little among the tissues tested (**Figure 1B**).

The biochemical characteristics and biological functions of PHO1 and PHO2 proteins have been investigated in many plants. The present study focused on analysis of the function of the *LjPHO3* gene in *L. japonicus*.

### Plastid Localization and Expression of LjPHO3 in *E. coli*

**FIGURE 2:**
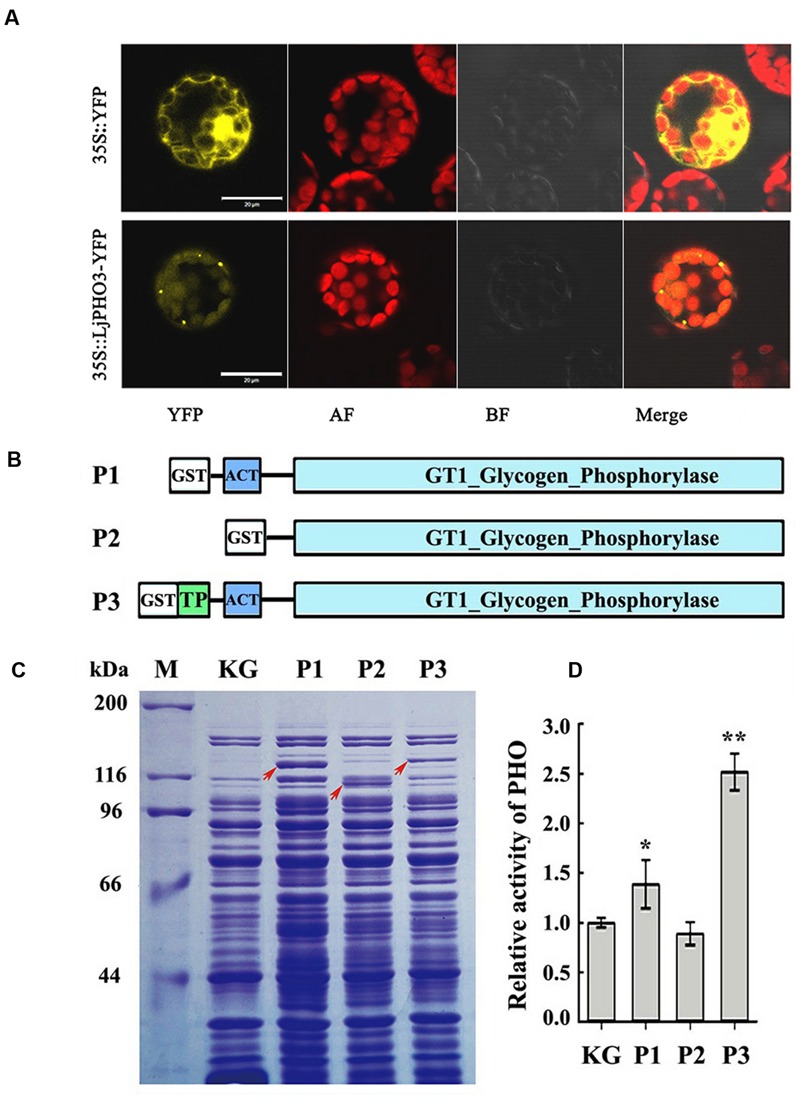
**Chloroplast localization of LjPHO3-eYFP and expression of LjPHO3 in *Escherichia coli*. (A)** Chloroplast localization of LjPHO3-eYFP. Protoplasts prepared from *Arabidopsis* leaves were used for gene transformation and observed by confocal laser scanning microscopy. Bar = 20 μm; **(B)** Simplified graphical representation of expression of truncated *LjPHO3* genes in *E. coli*; **(C)** SDS-PAGE of bacterially expressed LjPHO3 proteins. *E. coli* Rosetta was transformed with the native plasmid pGEX-KG or with reconstructed sequences (P1, P2, and P3), lyzed, and the lysate was analyzed for PHO activity. A protein standard ladder (marker) is indicated on the right. The gel was stained with Coomassie Brilliant Blue R-250 dye; **(D)** Starch phosphorolysis activity assay. Assays of the starch phosphorolysis activity of PHO were carried out at 28°C, using oyster glycogen as substrate. The data represent averages of three replicates with mean standard deviations. (Duncan test: ^∗^*P* < 0.05; ^∗∗^*P* < 0.01.)

To confirm the plastid localization of LjPHO3, as predicted by ChloroP 1.1, we examined the localization of LjPHO3-eYFP by laser scanning confocal microscopy. In P35S:LjPHO3-eYFP cells, eYFP fluorescence largely overlapped with the red fluorescence resulting from chlorophyll within chloroplasts (**Figure 2A**). This result suggested that LjPHO3 should be located in plastids. The strong fluorescent spots (**Figure 2A**) suggested the LjPHO3 proteins could also be deposited into protein bodies in the *Arabidopsis* cells.

*LjPHO3* was cloned and expressed in *E. coli* Rosetta to determine whether this *PHO* gene encoded an authentic PHO enzyme. The recombinant forms of PHO3 so produced include P1 (^Δ41^PHO, deletion of the putative TP domain sequences), P2 (^Δ183^PHO, in which the putative TP domain and ACT domain sequences were deleted), and P3 (the full length coding domain; **Figure 2B**). After induction of expression by IPTG, starch phosphorolysis activities of the total soluble protein in *E. coli* cells that contained recombinant P3 (full length) were on average 2.5-fold greater than the basal activity in *E. coli* cells containing the native plasmid (**Figures 2C,D**). To further study the enzymatic characteristics of LjPHO3, we purified the recombinant PHO3 proteins of P1 and P3 using the GST^⋅^BINDTM Resin. Unfortunately, neither starch phosphorolysis nor SS activity could be detected for any of the purified recombinant PHO3 proteins.

### Overexpression of the *LjPHO3* Gene Decreased Starch Accumulation in *L. japonicus* Leaves

**FIGURE 3:**
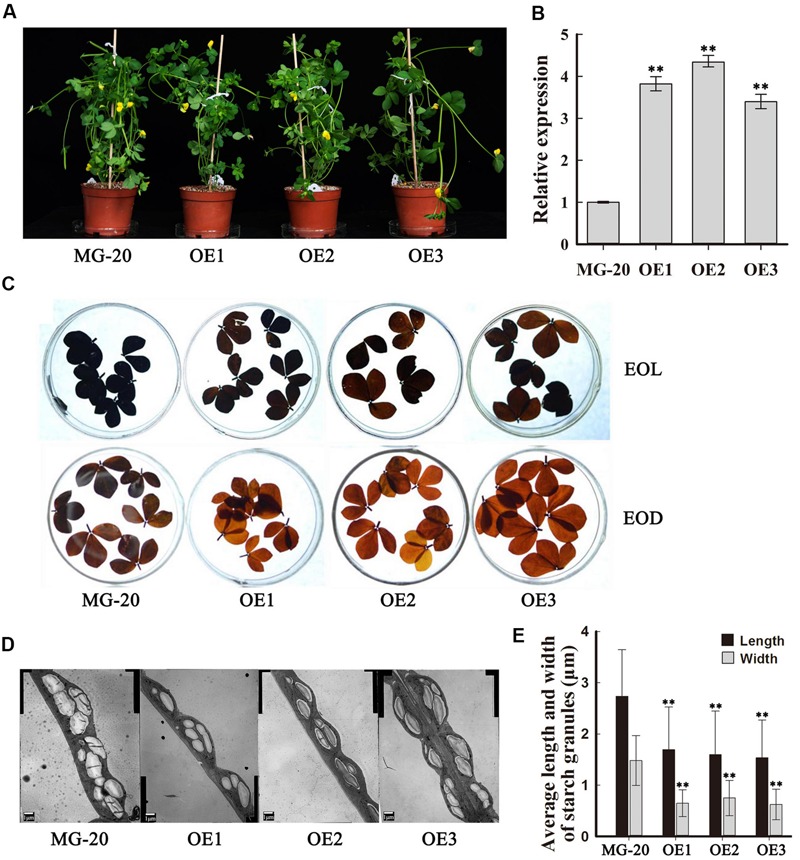
**Phenotype of transgenic plants carrying *LjPHO3* overexpression constructs.** MG-20, wild-type *L. japonicus*; OE1, OE2, and OE3, three different transgenic lines. **(A)** Ten-week-old seedlings grown in a growth chamber; **(B)** Expression analysis of the *LjPHO3* gene in leaves of 4-week-old seedlings. Values represent means of *n* = 6 ± SD (Duncan test: ^∗^*P* < 0.05; ^∗∗^*P* < 0.01); **(C)** Accumulation of starch in leaves of 10-week old seedlings. Leaves were heated in 80% (v/v) aqueous ethanol to remove pigments before staining with iodine (Lugol’s solution). EOL, end of light; EOD, end of dark; **(D)** Electron microscopic observation of starch granules; **(E)** Average length and width of starch granules (SGs). Over 500 SGs were measured for each line (**Supplementary Figure S3**). Values represent means of *n* ≥ 500 ± SD. (Duncan test: ^∗^*P* < 0.05; ^∗∗^*P* < 0.01.)

To investigate the function of *PHO3* in *L. japonicus*, the gene was overexpressed in the MG-20 variety under the control of the CaMV 35S promoter. Three independent overexpression of *LjPHO3* transgenic lines (*LjPHO3*-*OE1, 2*, and *3*) were established for use in these experiments (**Figure 3A**). Changes in the levels of *LjPHO3* transcripts were analyzed by qRT-PCR (**Figure 3B**).

Because of the predicted roles of PHO proteins in starch and oligosaccharide synthesis and/or degradation, we first measured the starch content in leaves of 10-week-old seedlings. The results showed that *LjPHO3*-*OE* leaves have an observable decrease in amounts of starch at both the end of the light period (EOL) and the end of the dark period (EOD; **Figure 3C**). Electron microscopic observation indicated that the starch granules at EOL were both shorter and narrower in *LjPHO3*-*OE* leaves than in wild-type leaves (**Figures 3D,E**; **Supplementary Figure S3B**).

**FIGURE 4:**
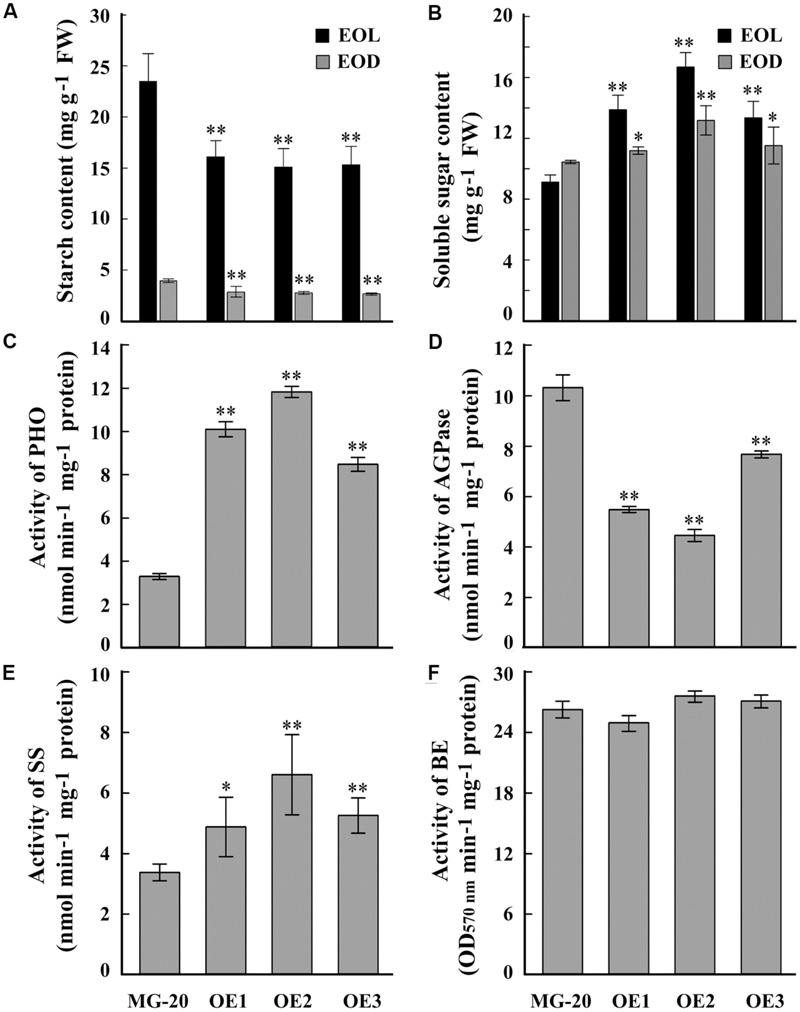
**Differences in biochemical indexes between wild-type leaves and *LjPHO3-OE* leaves.** MG-20, wild-type *L. japonicus*; OE1, OE2, and OE3, three different transgenic lines. The leaves were taken from 10-week-old seedlings grown in a growth chamber. **(A,B)** Starch and soluble sugar contents. EOL, end of light; EOD, end of dark; **(C)** Starch phosphorolysis activity of PHO; **(D**–**F)** Activities of ADP-Glucose pyrophosphorylase (AGPase), **(D)** Starch synthase (SS), **(E)** Starch branching enzyme (BE), **(F)** The data represent averages of three biological replicates with mean standard deviations. (Duncan test: ^∗^*P* < 0.05; ^∗∗^*P* < 0.01.)

A quantitative assay indicated that the starch content of *LjPHO3-OE* leaves was about 30% less than that of wild-type leaves (**Figure 4A**). On the other hand, the soluble sugar content was higher in *LjPHO3*-*OE* leaves than in wild-type leaves (**Figure 4B**). Next, we tested the activities of PHO and three SS enzymes in *LjPHO3*-*OE* and wild-type leaves. The results showed that the *LjPHO3*-*OE* leaves have higher PHO starch phosphorolysis activity and SS activity, but lower AGPase activity, than the wild-type leaves (**Figures 4C–E**). No significant difference in BE activity (**Figure 4F**), nor changes in zymogram bands corresponding to PHO SS activity (**Supplementary Figure S3C**), were observed between the *LjPHO3*-*OE* and wild-type leaves.

### Overexpression of the *LjPHO3* Gene Influences the Growth of *L. japonicus* Plants

**FIGURE 5:**
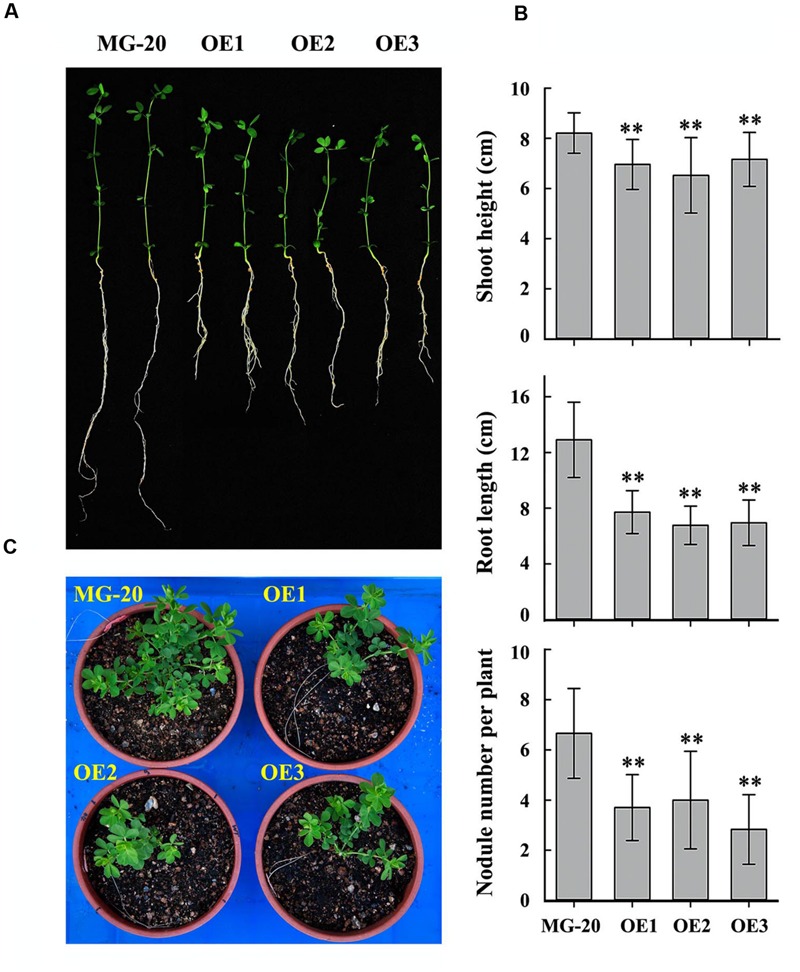
**Differences in plant size and nodule number between wild-type and *LjPHO3-OE* seedlings.** MG-20, wild-type *L. japonicus*; OE1, OE2, and OE3, three different transgenic lines. **(A)** Four-week-old seedlings grown in a growth chamber; **(B)** Differences in shoot height, main root length, and nodule number between wild-type and *LjPHO3-OE* seedlings. The data represent averages of 30–40 seedlings with mean standard deviations (Duncan test: ^∗^*P* < 0.05; ^∗∗^*P* < 0.01.); **(C)** Ten-week-old seedlings grown in a greenhouse under natural daylight.

Seeds of the *LjPHO3*-*OE* and wild-type lines were germinated, inoculated with *M. loti MAFF303099*, and grown on a vermiculite mixture for 4 weeks in a growth chamber (**Figure 5A**). The *LjPHO3*-*OE* seedlings were small with relatively shorter shoots and roots, and had fewer nodules, as compared to the wild-type seedlings (**Figure 5B**). At a later stage (10 weeks) under the greenhouse conditions, *LjPHO3*-*OE* seedlings were also small in comparison to the wild-type plants (**Figure 5C**), but there was no significant difference in flowering time. Their siliques were shorter, but their seeds were larger and heavier than those of wild-type plants (**Supplementary Figure S4**).

**FIGURE 6:**
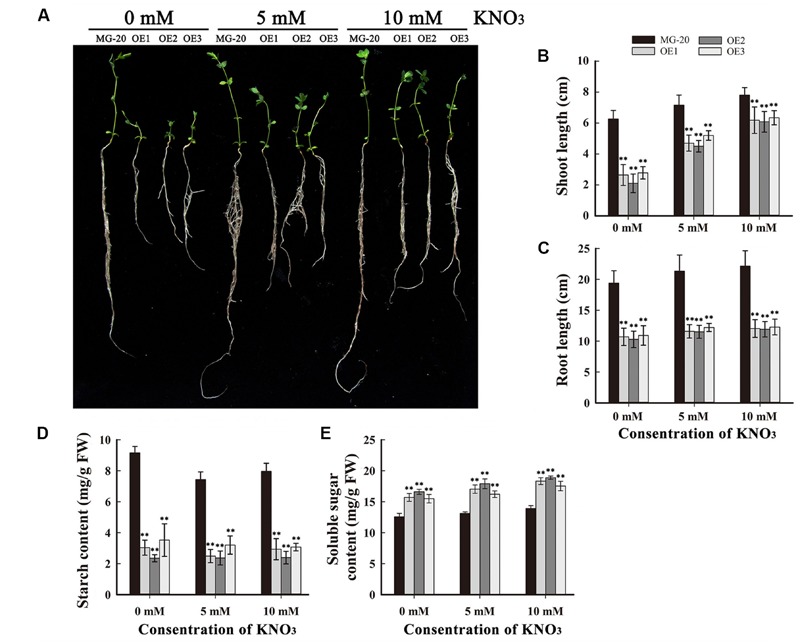
**Analysis of the effect of different levels of N-supply on the growth of *LjPHO3-OE* seedlings.** MG-20, wild-type *L. japonicus*; OE1, OE2, and OE3, three different transgenic lines. **(A)** Four-week-old seedlings grown under different N-supply conditions (0, 5, and 10 mM KNO_3_, respectively); **(B,C)** Shoot height **(B)** and main root length **(C)** of seedlings grown under different N-supply conditions; **(D,E)** Starch **(D)** and soluble sugar **(E)** contents of leaves at the end of the light period. The data represent averages of three biological replicates with mean standard deviations. (Duncan test: ^∗^*P* < 0.05; ^∗∗^*P* < 0.01.)

To study the influence of nitrogen on growth and starch accumulation in these plants, *LjPHO3*-*OE* and wild-type plants were germinated and grown on vermiculite (irrigated with Broughton and Dilworth nutrient solution containing 0, 5, and 10 mM KNO_3_, respectively) in transparent containers in a growth chamber. After 4 weeks, we observed that the *LjPHO3*-*OE* seedlings were small compared to the wild-type seedlings, especially under N-deficiency conditions (**Figures 6A–C**). The *LjPHO3*-*OE* leaves also had lower starch content (**Figure 6D**), but higher soluble sugar content (**Figure 6E**), than wild-type leaves under all three N-supply conditions.

### Overexpression of the *LjPHO3* Gene Decreased Pollen Fertility in *L. japonicus*

**FIGURE 7:**
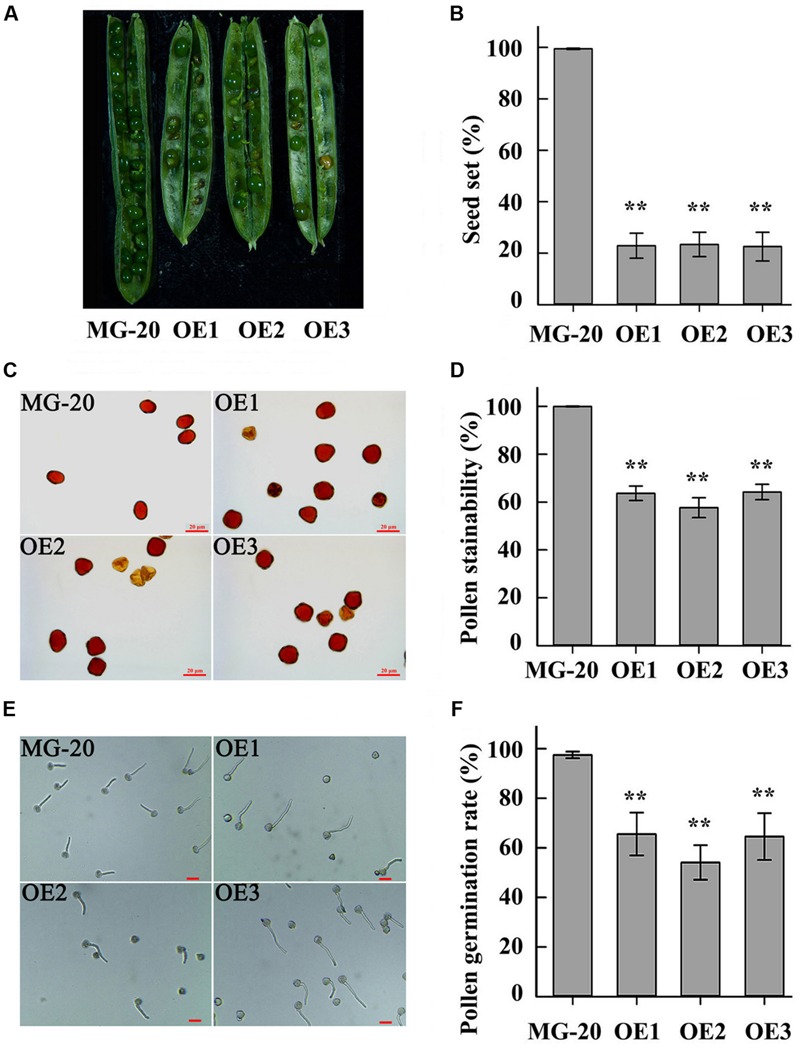
**Fertility changes in *LjPHO3-OE* plants.** MG-20, wild-type *L. japonicus*; OE1, OE2, and OE3, three different transgenic lines. **(A)** Silique filling stage and seeds; **(B)** Rate of seed set. The data represent averages of over 50 siliques with mean standard deviations (Duncan test: ^∗^*P* < 0.05; ^∗∗^*P* < 0.01.); **(C)** Morphology of pollen grains; **(D)** Stainability of pollen by iodine–potassium iodide solution; **(E)** *In vitro* pollen germination test. Scale bar = 20 μm; **(F)** Pollen germination rate. For each plant line, pollen grains from 5 to 6 anthers of each of 6 to 8 plants were analyzed. (Duncan test: ^∗^*P* < 0.05; ^∗∗^*P* < 0.01.) Scale bar = 20 μm.

Compared to the wild-type, *LjPHO3*-*OE* plants displayed a 75% reduction in rate of seed set (**Figures 7A,B**). Wild-type pollen grains were round with ample cytoplasm, whereas many pollen grains from *LjPHO3*-*OE* plants were shrunken, irregular, and wizened, with scant cytoplasm (**Figure 7C**). The proportion of pollen grains from wild-type plants that became dark blue upon iodine staining was close to 100%, while the proportion from *LjPHO3*-*OE* plants was around 60% (**Figure 7D**). After incubation on medium for 30 min at 37°C, the germination rate of pollen from *LjPHO3-OE* plants was around 60%, whereas the rate of germination for wild-type pollen was about 97% (**Figures 7E,F**). It is clear that overexpression of *LjPHO3* substantially reduces pollen fertility in *L. japonicus*.

## Discussion

Plastids are descended from a cyanobacterial symbiosis and starch metabolism genes probably coevolved following at least two rounds of whole genome duplication during the early period of plant evolution (Deschamps et al., 2008). It is therefore possible in principle for any plant lineage to have retained three isoforms of each starch metabolism protein. Only two starch phosphorylase isoforms, the plastidic starch phosphorylase (PHO1/PHOL) and the cytosolic starch phosphorylase (PHO2/PHOH), have previously been reported in higher plants (Deschamps et al., 2008). We detected a new starch phosphorylase family protein in some plants, which form a new subclade together with two *PHO* genes from the green algae *O. lucimarinus* and *C. reinhardtii* (*PHOB/STA4*) on the phylogenetic tree (Wu et al., 2015). In this study, we observed that the *PHO3* gene was present in the genome of *L. japonicus* (**Supplementary Figure S1**) and it participates in transitory starch metabolism in *L. japonicus* leaves. These results indicate the *PHO3* gene should be lost in some plant linkages during their evolution. The loss of genes encoding other SS enzymes was also observed in some plants. For examples, cruciferae plants such as *Arabidopsis* and *Brassica rapa* don’t have the gene encoding BE I isoform in their genomes (Dumez et al., 2006). Many plants lost the gene encoding SS VI isoform although its biological function is unknown (Wu et al., 2015). In these plants, the biological functions of these genes may be lost, or replaced by other genes during their evolution.

The PHOB/STA4 protein is located in the plastid in *C. reinhardtii* (Dauvillée et al., 2006). In *Arabidopsis* protoplasts, eYFP fluorescence of LjPHO3-eYFP largely overlapped with the red chlorophyll fluorescence within chloroplasts (**Figure 2A**), which indicates that LjPHO3 is also a plastidic protein. Although the crude enzyme extract from *E. coli* cells containing the recombinant LjPHO3 protein showed higher starch phosphorolysis activity (**Figure 2C**), purified recombinant LjPHO3 proteins did not show any SS or starch phosphorolysis activities using the substrates glucose-1-phosphate, and/or maltose, maltotriose, maltoheptaose, glycogen, amylopectin, and amylose. The catalytic properties of the LjPHO3 protein therefore remain to be determined.

In *C. reinhardtii*, the *sta4* mutants were found as mutants expressing a conditional low-starch high-amylose phenotype only in conditions of high polysaccharide synthesis (Libessart et al., 1995; Dauvillée et al., 2006). The authors speculated that the role of PHOB might be indirect through its involvement in a multienzyme complex (such as the BE–phosphorylase complex) selectively active during starch biosynthesis under high carbon flux (Dauvillée et al., 2006). The majority of ACT domain-containing proteins appears to interact with amino acids and is involved in some aspect of regulation of amino acid metabolism (Grant, 2006). So, it could not exclude the possibility that one or more amino acids may be involved in the regulation of the activity of PHOB in starch turnover in *C. reinhardtii*.

Like the *C. reinhardtii* PHOB/STA4, the LjPHO3 protein has an ACT domain [cl09141] in the N-terminus, but it lacks the L78 domain which is present in PHO1 proteins (**Figure 1A**; **Supplementary Figure S2**). In this study, we observed that the LjPHO3 proteins appeared to play a negative role in starch accumulation in *L. japonicus* leaves. Overexpression of *LjPHO3* in *L. japonicus* resulted in a significant decrease in amounts of starch and sizes of starch granules but an increased amount of soluble sugars in leaves of the transgenic plants (**Figures 3C** and **4A,B**; **Supplementary Figure S3**). This effect was not affected by nitrogen supplying levels (**Figures 6D,E**), but the growth of *LjPHO3*-*OE* seedlings was seriously retarded under N-deficiency conditions (**Figure 6A**). These results implied that the increased activity of phosphorylase (**Figure 4C**) increased the phosphorolysis of the transitory starches in the *LjPHO3*-*OE* leaves. The increase of SS activity (**Figure 4E**) may be induced by the elevated content of sugars in *LjPHO3*-*OE* leaves (**Figure 4B**). In rice and *Arabidopsis*, the expression levels of *SS* genes, but not *AGPase* genes those function in leaves have been reported to be upregulated by higher sugars in leaves (Dian et al., 2003, 2005; Niittylä et al., 2004; Akihiro et al., 2005). Reduction in starch accumulation may elevate sugar export from chloroplasts further led to the increase of Pi content in chloroplasts. In leaves of higher plants, the activity of AGPase is mainly modified at protein level, such as regulated by 3-phosphoglycerate (activator) and inorganic orthophosphate (inhibitor), and also redox modification (Ballicora et al., 2004). The reduction of AGPase activity in *LjPHO3-OE* leaves (**Figure 4D**) may be due to changes in Pi content and sugar (-P) pool in chloroplasts. The detail changes in photosynthetic parameters, sugar/sugar-P composition and other compounds between the wild-type and transgenic leaves remained to be determined next. It has been reported that transitory starch is produced in the chloroplast of leaves during the day, and degrades to support metabolism and growth at night in many plants (Smith and Stitt, 2007; Vriet et al., 2010). In consequence, the functional transitory starch turnover is crucial for normal growth of plants. The smaller size of *LjPHO3-OE* seedlings may be due to an insufficiency of sugars supplied by the leaves at night (**Figures 5A,C** and **6A**). The reduction in pollen fertility (**Figure 7**) may be due to the decreased in deposition of starch in developing pollen grains of *LjPHO3-OE* plants. Alternatively, it may be because of the insufficient amount of sugars supplied by the leaves. As a result of so many of the pollen grains being sterile, seed set was reduced in *LjPHO3-OE* plants (**Figure 7**).

Considering the phenotype of the overexpression lines it was logical to test the effect of *LjPHO3* knock-down. Two different approaches were used, including RNA interference lines (MG-20) and two LTR retrotransposon insertion lines (Gifu; DK02: No.30006161 and DK07: No.30056666 from the LORE1 insertion mutant resource^14^). Sadly, *LjPHO3* expression was not reduced significantly in the interference lines and those plants did not exhibit related phenotypes. Furthermore, the two tested insertion lines, were not knock-out mutants, as full length transcript cDNA was detected in homozygote plants (data not shown). It would be interesting to test the newly available insertion lines to conclude on the effect of *LjPHO3* knock-out.

Starch synthesis or starch degradation? The plastidial PHO1 may play different physiological role between species, growing conditions and tissues, and one or more unknown factors were involved in regulating the action (Zeeman et al., 2004; Satoh et al., 2008; Lin et al., 2012; Streb and Zeeman, 2012; Malinova et al., 2014). The same assumption could also apply to the physiological role of the PHO3 protein. In *C. reinhardtii*, PHOB/PHO3 may play a role in storage starch biosynthesis in conditions of nitrogen starvation (Dauvillée et al., 2006). In *L*. *japonicus*, PHO3 should participate in starch degradation in leaves based on the present results. On the other hand, overexpression of the *LjPHO3* gene in rice did not significantly affect the starch content of either leaves or endosperms, or the rate of seed set rate in transgenic rice plants (data not shown). These results imply that one or more additional factor (s) in *L. japonicus* plants are required for the PHO3 protein to be active in regulating starch accumulation. They could also explain why the purified recombinant PHO3 proteins showed no SS or starch phosphorolysis activity *in vitro*.

## Conclusion

*Lotus japonicus* has a gene encoding the PHO3 isoform. The LjPHO3 protein lacks the L78 domain but has an ACT domain, and it is located in the chloroplast. Overexpression of *LjPHO3* in *L. japonicus* results in a reduction in starch content in leaves. The reduction in starch deposition has a major impact on plant growth, pollen fertility, and rate of seed set rate in the transgenic plants. However, the catalytic properties of the LjPHO3 protein remain to be further studied. To our knowledge, the results presented here represent the first data on the function of the PHO3 protein subfamily in higher plants.

## Author Contributions

The research was designed by HJ, GW, YC, ML, and PW. The experiments were performed by SQ, YT, and the data were analyzed by SQ. The manuscript was written by SQ.

## Conflict of Interest Statement

The authors declare that the research was conducted in the absence of any commercial or financial relationships that could be construed as a potential conflict of interest.

The reviewer AT and handling Editor declared their shared affiliation, and the handling Editor states that the process nevertheless met the standards of a fair and objective review.

## Footnotes

1 http://www.ncbi.nlm.nih.gov/

2 http://www.kazusa.or.jp/lotus/blast.html

3 http://www.ncbi.nlm.nih.gov/Structure/bwrpsb/bwrpsb.cgi

4 http://www.cbs.dtu.dk/services/TargetP/

5 http://www.merckmillipore.com/

6 http://www.qiagen.com

7 http://www.roche.com

8 http://au.promega.com

9 http://primer3.ut.ee/

10 http://www.zeiss.com/

11 http://www.jeol.com

12 http://www.kazusa.or.jp/lotus/blast.html

13 www.cbs.dtu.dk/services/ChloroP/

14 http://lotus.au.dk

## Supplementary Material

FIGURE S1 **Neighbor-joining unrooted tree.** Bootstrap values were calculated for 100 replicates, and values are indicated at the corresponding nodes. The branch length corresponding to the number of substitutions per site is given and the database accession numbers of sequences are indicated in brackets.

FIGURE S2 **Comparison of the deduced amino acid sequences of PHO proteins from *Lotus japonicus* and *Chlamydomonas*.** Conserved amino acids are indicated by shaded squares.

FIGURE S3 **Phenotype of starch granules and starch phosphorylase isoforms in wild-type leaves and *LjPHO3-OE* leaves. (A)** Electron microscopic observation of starch granules. More chloroplasts lacked starch granules in *LjPHO3-OE* plants than in wild-type plants; **(B)** Distribution of the lengths and widths of measured starch granules; **(C)** Zymogram analysis of starch synthesis activities of the PHO enzymes. Soluble proteins from crude extracts of leaves were subjected to native PAGE [7.5% (w/v) acrylamide slab gel containing 0.5% (w/v) oyster glycogen] and stained to reveal phosphorylase isoforms.

FIGURE S4 **Differences in silique length and seed size between MG-20 and *LjPHO3-OE* seedlings. (A)** and **(B)** Difference in siliques length between MG-20 and *LjPHO3-OE* seedlings; **(C)** Seed size; **(D)** The 1000-seed weight. Values represent means of *n* = 6 ± SD. (Duncan test: ^∗^*P* < 0.05; ^∗∗^*P* < 0.01.)

## References

[B1] Akihiro T. Mizuno K. Fujimura T. (2005). Gene expression of ADP-glucose pyrophosphorylase and starch contents in rice cultured cells are cooperatively regulated by sucrose and ABA. *Plant Cell Physiol.* 46 937–946. 10.1093/pcp/pci101 15821022

[B2] Ballicora M. A. Iglesias A. A. Preiss J. (2004). ADP-Glucose pyrophosphorylase: a regulatory enzyme for plant starch synthesis. *Photosynth. Res.* 79 1–24. 10.1023/B:PRES.0000011916.67519.58 16228397

[B3] Chen H. M. Chang S. C. Wu C. C. Cuo T. S. Wu J. S. Juang R. H. (2002). Regulation of the catalytic behaviour of L-form starch phosphorylase from sweet potato roots by proteolysis. *Physiol. Plant* 114 506–515. 10.1034/j.1399-3054.2002.1140402.x 11975723

[B4] Chen Y. P. Chen W. Li X. L. Jiang H. W. Wu P. Z. Xia K. F. (2014). Knockdown of LjIPT3 influences nodule development in *Lotus japonicus*. *Plant Cell Physiol.* 55 183–193. 10.1093/pcp/pct171 24285753

[B5] Dauvillée D. Chochois V. Steup M. Haebel S. Eckermann N. Ritte G. (2006). Plastidial phosphorylase is required for normal starch synthesis in *Chlamydomonas reinhardtii*. *Plant J.* 48 274–285. 10.1111/j.1365-313X.2006.02870.x 17018036

[B6] Deschamps P. Moreau H. Worden A. Z. Dauvillée D. Ball S. G. (2008). Early gene duplication within Chloroplastida and its correspondence with relocation of starch metabolism to chloroplasts. *Genetics* 178 2373–2387. 10.1534/genetics.108.087205 18245855 PMC2323822

[B7] Dian W. Jiang H. Chen Q. Liu F. Wu P. (2003). Cloning and characterization of the granule-bound starch synthase II gene in rice, gene expression is regulated by the nitrogen level, sugar and circadian rhythm. *Planta* 218 261–268. 10.1007/s00425-003-1101-9 12955512

[B8] Dian W. Jiang H. Wu P. (2005). Evolution and expression analysis of starch synthase III and IV in rice. *J. Exp. Bot.* 56 623–632. 10.1093/jxb/eri065 15642712

[B9] DuBois M. Gilles K. Hamilton J. K. Rebers P. A. Smith F. (1951). A colorimetric method for the determination of sugars. *Nature* 168 167–167. 10.1038/168167a0 14875032

[B10] Dumez S. Wattebled F. Dauvillee D. Delvalle D. Planchot V. Ball S. G. (2006). Mutants of *Arabidopsis* lacking starch branching enzyme II substitute plastidial starch synthesis by cytoplasmic maltose accumulation. *Plant Cell* 18 2694–2709. 10.1105/tpc.105.037671 17028209 PMC1626616

[B11] Emanuelsson O. Nielsen H. von Heijne G. (1999). ChloroP, a neural network-based method for predicting chloroplast transit peptides and their cleavage sites. *Protein Sci.* 8 978–984. 10.1110/ps.8.5.978 10338008 PMC2144330

[B12] Fettke J. Chia T. Eckermann N. Smith A. Steup M. (2006). A transglucosidase necessary for starch degradation and maltose metabolism in leaves at night acts on cytosolic heteroglycans (SHG). *Plant J.* 46 668–684. 10.1111/j.1365-313X.2006.02732.x 16640603

[B13] Fettke J. Poeste S. Eckermann N. Tiessen A. Pauly M. Geigenberger P. (2005). Analysis of cytosolic heteroglycans from leaves of transgenic potato (*Solanum tuberosum* L.) plants that under- or overexpress the Pho 2 phosphorylase isozyme. *Plant Cell Physiol.* 46 1987–2004. 10.1093/pcp/pci214 16230332

[B14] Grant G. A. (2006). The ACT domain: a small molecule binding domain and its role as a common regulatory element. *J. Biol. Chem.* 281 33825–33829. 10.1074/jbc.R600024200 16987805

[B15] Hwang S. K. Singh S. Cakir B. Satoh H. Okita T. W. (2016). The plastidial starch phosphorylase from rice endosperm: catalytic properties at low temperature. *Planta* 243 999–1009. 10.1007/s00425-015-2461-7 26748915

[B16] Jiang H. Dian W. Wu P. (2003). Effect of high temperature on fine structure of amylopectin in rice endosperm by reducing the activity of the starch branching enzyme. *Phytochemistry* 63 53–59. 10.1016/S0031-9422(03)00005-0 12657298

[B17] Jiang H. W. Li M. R. Liang N. T. Yan H. B. Wei Y. B. Xu X. L. (2007). Molecular cloning and function analysis of the stay green gene in rice. *Plant J.* 52 197–209. 10.1111/j.1365-313X.2007.03221.x 17714430

[B18] Kruger N. J. ap Rees T. (1983). Properties of α-glucan phosphorylase from pea chloroplasts. *Phytochemistry* 22 1891–1898. 10.1016/0031-9422(83)80007-7

[B19] Libessart N. Maddelein M. L. Koornhuyse N. Decq A. Delrue B. Mouille G. (1995). Storage, photosynthesis, and growth: the conditional nature of mutations affecting starch synthesis and structure in *Chlamydomonas*. *Plant Cell* 7 1117–1127. 10.1105/tpc.7.8.1117 12242401 PMC160938

[B20] Lin Y. C. Chen H. M. Chou I. M. Chen A. N. Chen C. P. Young G. H. (2012). Plastidial starch phosphorylase in sweet potato roots is proteolytically modified by protein-protein interaction with the 20S proteasome. *PLoS ONE* 7:e35336 10.1371/journal.pone.0035336 PMC3323651 22506077

[B21] Lu Y. Steichen J. M. Yao J. Sharkey T. D. (2006). The role of cytosolic alpha-glucan phosphorylase in maltose metabolism and the comparison of amylomaltase in *Arabidopsis* and *Escherichia coli*. *Plant Physiol.* 142 878–889. 10.1104/pp.106.086850 16980562 PMC1630732

[B22] Malinova I. Mahlow S. Alseekh S. Orawetz T. Fernie A. R. Baumann O. (2014). Double knockout mutants of *Arabidopsis* grown under normal conditions reveal that the plastidial phosphorylase isozyme participates in transitory starch metabolism. *Plant Physiol.* 164 907–921. 10.1104/pp.113.227843 24302650 PMC3912115

[B23] Marchler-Bauer A. Derbyshire M. K. Gonzales N. R. Lu S. Chitsaz F. Geer L. Y. (2015). CDD, NCBI’s conserved domain database. *Nucleic Acids Res.* 43 D222–D226. 10.1093/nar/gku1221 25414356 PMC4383992

[B24] Mori H. Tanizawa K. Fukui T. (1993). A chimeric alpha-glucan phosphorylase of plant type L and H isozymes. Functional role of 78-residue insertion in type L isozyme. *J. Biol. Chem.* 268 5574–5581. 8449920

[B25] Niittylä T. Messerli G. Trevisan M. Chen J. Smith A. M. Zeeman S. C. (2004). A previously unknown maltose transporter essential for starch degradation in leaves. *Science* 303 87–89. 10.1126/science.1091811 14704427

[B26] Nishi A. Nakamura Y. Tanaka N. Satoh H. (2001). Biochemical and genetic analysis of the effects of amylose-extender mutation in rice endosperm. *Plant Physiol.* 127 459–472. 10.1104/pp.010127 11598221 PMC125082

[B27] Satoh H. Shibahara K. Tokunaga T. Nishi A. Tasaki M. Hwang S. K. (2008). Mutation of the plastidial α-glucan phosphorylase gene in rice affects the synthesis and structure of starch in the endosperm. *Plant Cell* 20 1833–1849. 10.1105/tpc.107.054007 18621947 PMC2518224

[B28] Smith A. M. Stitt M. (2007). Coordination of carbon supply and plant growth. *Plant Cell Environ.* 30 1126–1149. 10.1111/j.1365-3040.2007.01708.x 17661751

[B29] Streb S. Zeeman S. C. (2012). Starch metabolism in *Arabidopsis*. *Arabidopsis Book* 10:e0160 10.1199/tab.0160 PMC3527087 23393426

[B30] Tamura K. Dudley J. Nei M. Kumar S. (2007). MEGA4, molecular evolutionary genetics analysis (MEGA) software version 4.0. *Mol. Biol. Evol.* 24 1596–1599. 10.1093/molbev/msm092 17488738

[B31] Vriet C. Welham T. Brachmann A. Pike M. Pike J. Perry J. (2010). A Suite of *Lotus japonicus* starch mutants reveals both conserved and novel features of starch metabolism. *Plant Physiol.* 154 643–655. 10.1104/pp.110.161844 20699404 PMC2949007

[B32] Wirtz W. Stitt M. Heldt H. W. (1980). Enzymic determination of metabolites in the subcellular compartments of spinach protoplasts. *Plant Physiol.* 66 187–193. 10.1104/pp.66.1.187 16661385 PMC440555

[B33] Wu P. Z. Zhou C. P. Cheng S. F. Wu Z. Y. Lu W. J. Han J. (2015). Integrated genome sequence and linkage map of physic nut (*Jatropha curcas* L.), a biodiesel plant. *Plant J.* 81 810–821. 10.1111/tpj.12761 25603894

[B34] Yoo S. D. Cho Y. H. Sheen J. (2007). *Arabidopsis* mesophyll protoplasts: a versatile cell system for transient gene expression analysis. *Nat. Protoc.* 2 1565–1572. 10.1038/nprot.2007.199 17585298

[B35] Zeeman S. C. Thorneycroft D. Schupp N. Chapple A. Weck M. Dunstan H. (2004). Plastidial α-glucan phosphorylase is not required for starch degradation in *Arabidopsis* leaves but has a role in the tolerance of abiotic stress. *Plant Physiol.* 135 849–858. 10.1104/pp.103.032631 15173560 PMC514120

