# Supplementary material for: Overexpression of the Starch Phosphorylase-Like Gene (PHO3) in Lotus japonicus has a Profound Effect on the Growth of Plants and Reduction of Transitory Starch Accumulation
Source: Front Plant Sci. 2016 Aug 31;7:1315. doi: 10.3389/fpls.2016.01315 (PMC5005325; doi:10.3389/fpls.2016.01315)
Supplement: Supplementary file 1 [file Table_1.DOCX]

**Table S1.**

Primers used in this study.

| Gene ID | Sequence (5→`3`) | | PCR product length (bp) |
| --- | --- | --- | --- |
|  | Forward primer | Reverse primer |  |
| Cloning of cDNA (containing complete coding sequences) | | |  |
| LjPHO3 | ATCTCCTTAGATCAGAAAGGTAAAC | aactgcagTCAGAGAGGGCATCGGCAGGGATCA | 3109 |
| Subcellular localization | | |  |
| LjPHO3-F | tctcgagcATGCTAACCGTTTCGTTTCCT | gcggtaccgGAGAGGGCATCGGCAGGG | 2988 |
| Protein expression in *E. coli* | | |  |
| P1 | ggaattctagagCTCCGGAACTACAACAACCGTTT | ccctcgagTGAGAGGGCATCGGCAGGG | 2838 |
| P2 | catgccatggATGGGTTTCTGAAGAAT | ccctcgagTGAGAGGGCATCGGCAGGG | 2649 |
| P3 | ggaattctagagATGCTAACCGTTTCGTTTCCTC | ccctcgagTGAGAGGGCATCGGCAGGG | 2988 |
| qRT-PCR | | |  |
| LjPHO3 | TGGTTATACCAGGGGCTGAC | TCTCgAACCATCCTCAACACAC | 265 |
| LjPHO1 | TCTGGTTACGGTCGTGGTGATTACT | AATTGTGCGGTCACTGCTGAACTT | 242 |
| LjPHO2;1 | AATGGAGGCGAGTGGAACTAGCA | GGCACCAAAGAGGAAGAAGTTGTCT | 221 |
| LjPHO2;2 | AGGTTTGTTCCTCGGGTTTGTATT | CTTGTTCCACTGGCCTCCATC | 216 |
| Ubiquitin | ATGTGCATTTTAAGACAGGG | GAACGTAGAAGATTGCCTGAA | 148 |
