# Supplementary material for: Overexpression of the Starch Phosphorylase-Like Gene (PHO3) in Lotus japonicus has a Profound Effect on the Growth of Plants and Reduction of Transitory Starch Accumulation
Source: Front Plant Sci. 2016 Aug 31;7:1315. doi: 10.3389/fpls.2016.01315 (PMC5005325; doi:10.3389/fpls.2016.01315)
Supplement: Supplementary file 2 [file Table_2.DOCX]

**Table S2.**

| Subfamily | LjPHO1;1 | LjPHO1;2 | LjPHO2 | LjPHO3 |
| --- | --- | --- | --- | --- |
| Gene ID | Lj2g3v1079510 | Lj0g3v0360239 | LjB08M07.90.r2.m | Lj6g3v2006830 |
| Protein length (aa) | 973 | 976 | 846 | 996 |
| Expression (EST) | yes | yes | yes | yes |
| Exons (No./bp) |  |  |  |  |
| 1 | 360 (232)* | 226 (unknown) | 143 | 674(660)* |
| 2 | 239 | 230 | 37 | 115 |
| 3 | 102 | 102 | 253(225)* | 101 |
| 4 | 177 | 177 | 180 | 92 |
| 5 | 276 | 276 | 177 | 136 |
| 6 | 272 | 272 | 86 | 69 |
| 7 | 118 | 118 | 190 | 102 |
| 8 | 405 | 429 | 93 | 99 |
| 9 | 153 | 153 | 179 | 111 |
| 10 | 129 | 129 | 118 | 207 |
| 11 | 237 | 237 | 129 | 108 |
| 12 | 77 | 77 | 219 | 96 |
| 13 | 187 | 187 | 204 | 57 |
| 14 | 201 | 201 | 159 | 147 |
| 15 | 450 (117)** | 241 (117)** | 264 | 60 |
| 16 |  |  | 191 | 105 |
| 17 |  |  | 397 (127)** | 162 |
| 18 |  |  |  | 74 |
| 19 |  |  |  | 124 |
| 20 |  |  |  | 92 |
| 21 |  |  |  | 121 |
| 22 |  |  |  | 301 (153)** |

The exon/intron composition of *Lotus japonicus* starch phosphorylase genes.

* Location of the start codon in this exon; ** Location of the stop codon in this exon.
