# Supplementary figures and images for: Overexpression of the Starch Phosphorylase-Like Gene (PHO3) in Lotus japonicus has a Profound Effect on the Growth of Plants and Reduction of Transitory Starch Accumulation
Source: Front Plant Sci. 2016 Aug 31;7:1315. doi: 10.3389/fpls.2016.01315 (PMC5005325; doi:10.3389/fpls.2016.01315)

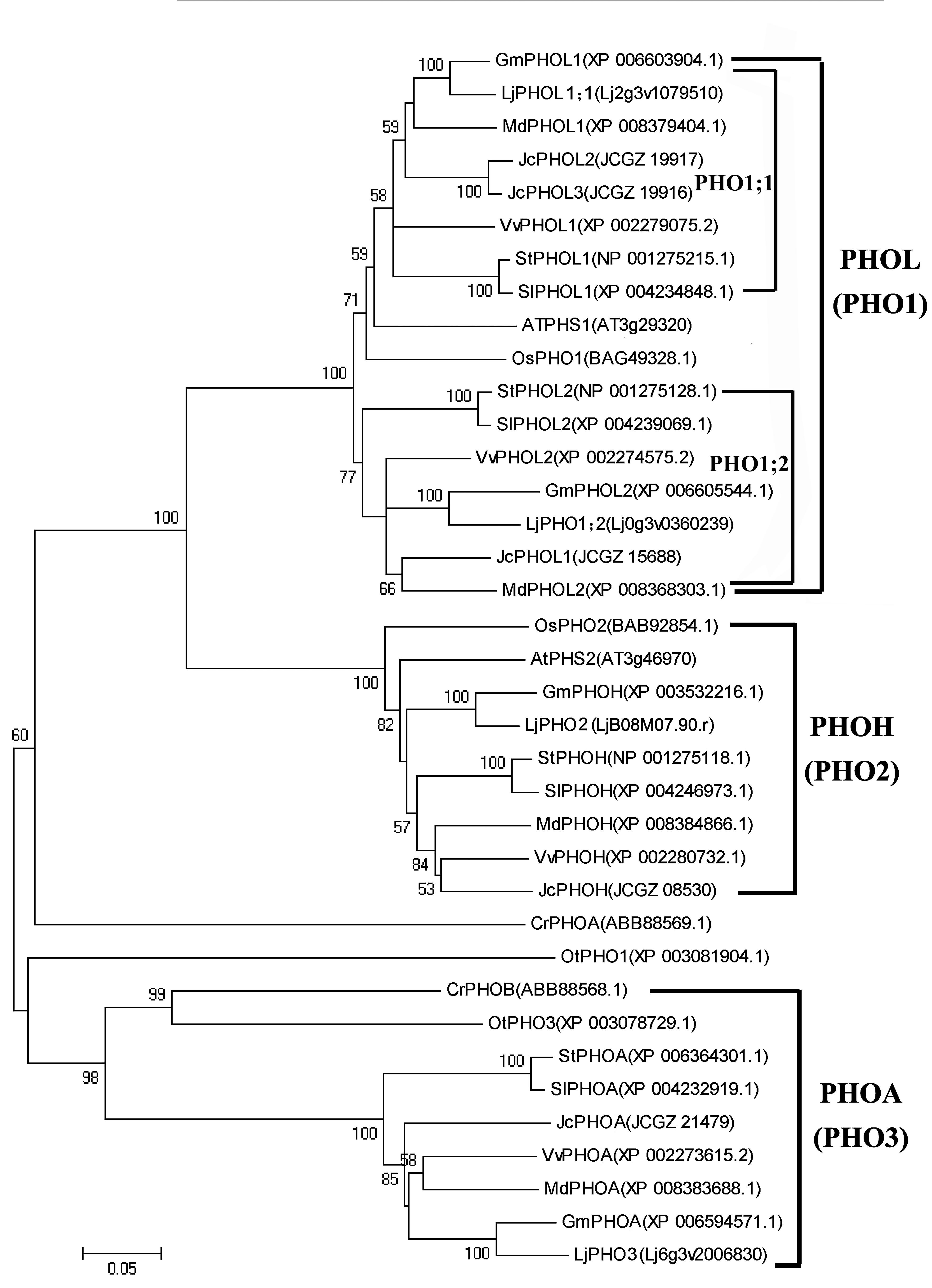

Supplement: FIGURE S1 — Neighbor-joining unrooted tree. Bootstrap values were calculated for 100 replicates, and values are indicated at the corresponding nodes. The branch length corresponding to the number of substitutions per site is given and the database accession numbers of sequences are indicated in brackets. [file Image_1.JPEG]

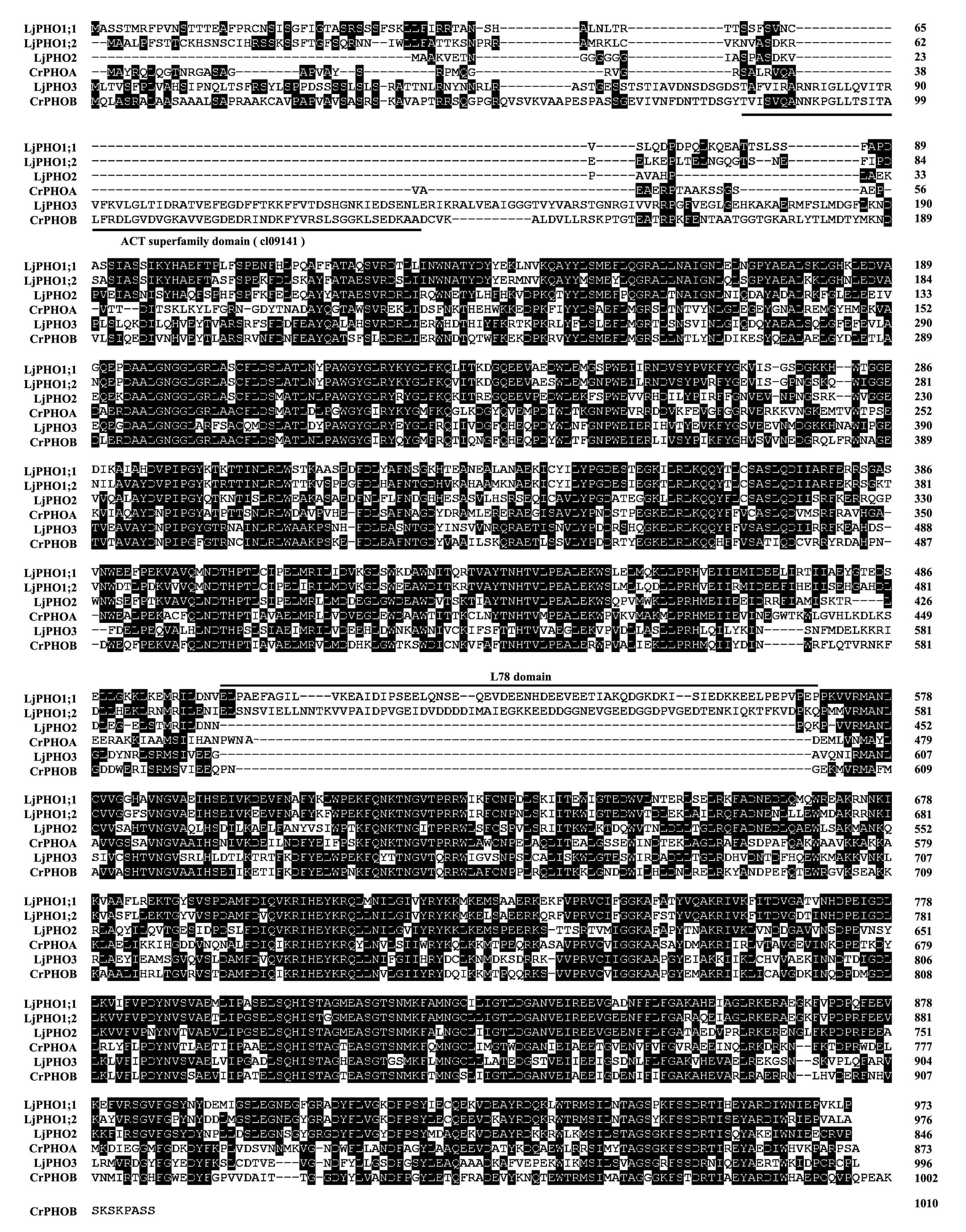

Supplement: FIGURE S2 — Comparison of the deduced amino acid sequences of PHO proteins from Lotus japonicus and Chlamydomonas. Conserved amino acids are indicated by shaded squares. [file Image_2.JPEG]

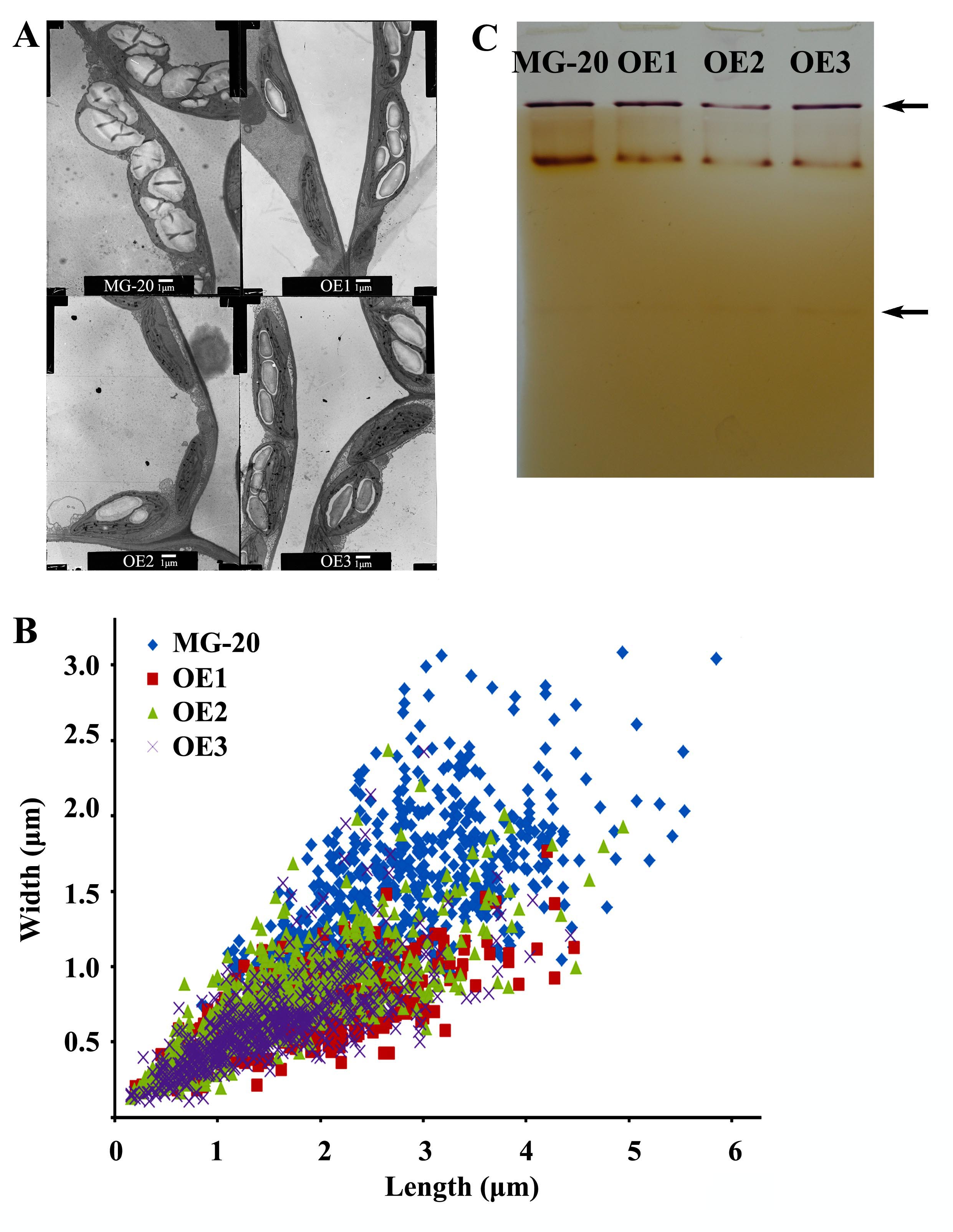

Supplement: FIGURE S3 — Phenotype of starch granules and starch phosphorylase isoforms in wild-type leaves and LjPHO3-OE leaves. (A) Electron microscopic observation of starch granules. More chloroplasts lacked starch granules in LjPHO3-OE plants than in wild-type plants; (B) Distribution of the lengths and widths of measured starch granules; (C) Zymogram analysis of starch synthesis activities of the PHO enzymes. Soluble proteins from crude extracts of leaves were subjected to native PAGE [7.5% (w/v) acrylamide slab gel containing 0.5% (w/v) oyster glycogen] and stained to reveal phosphorylase isoforms. [file Image_3.JPEG]

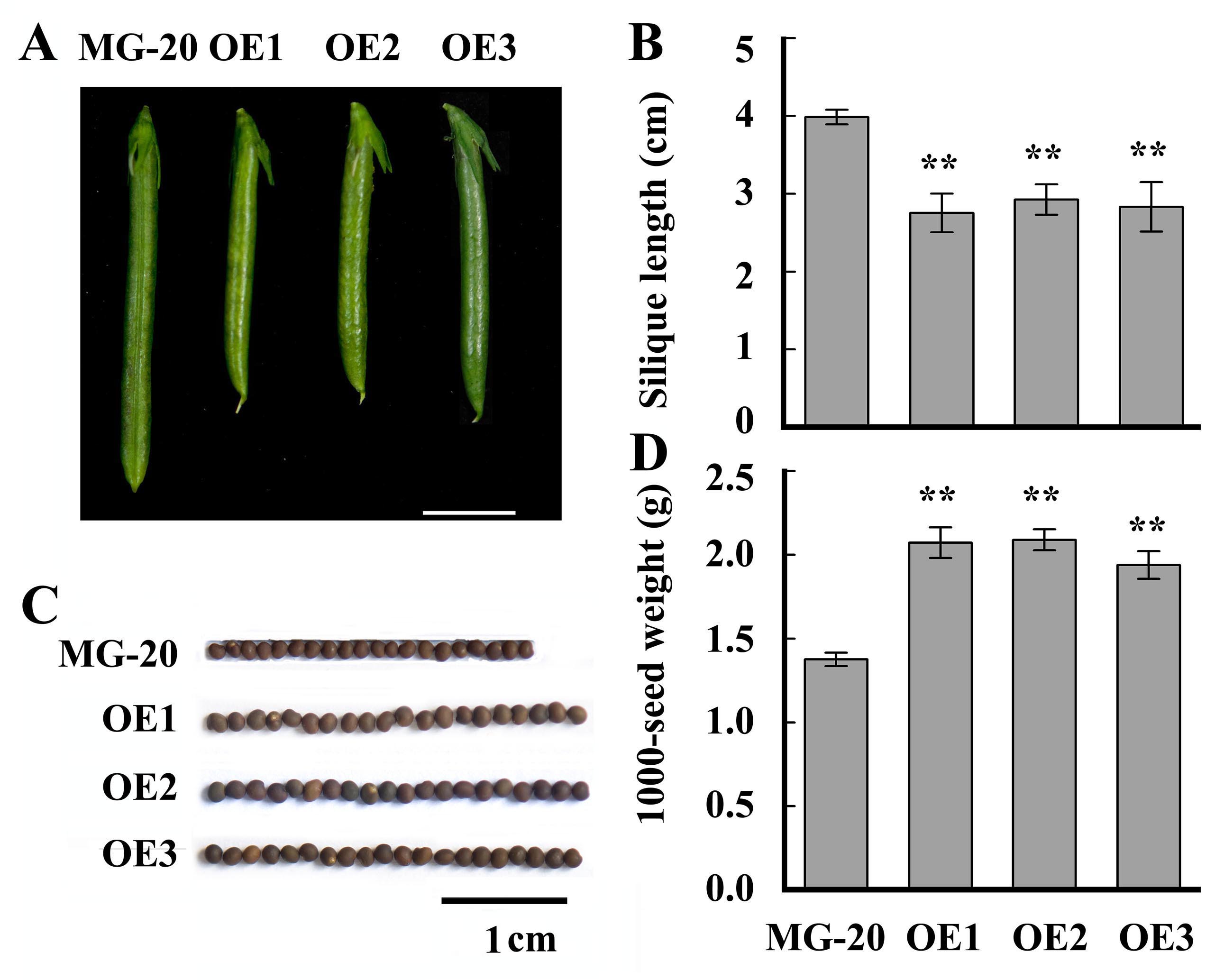

Supplement: FIGURE S4 — Differences in silique length and seed size between MG-20 and LjPHO3-OE seedlings. (A) and (B) Difference in siliques length between MG-20 and LjPHO3-OE seedlings; (C) Seed size; (D) The 1000-seed weight. Values represent means of n = 6 ± SD. (Duncan test: ∗P < 0.05; ∗∗P < 0.01.) [file Image_4.JPEG]
